# Experience in fostering regional collaboration and Coordination to use data for battling infectious diseases in sub-Saharan Africa

**DOI:** 10.24248/eahrj.v5i2.660

**Published:** 2021-11-15

**Authors:** Geoffrey Arunga, Tome Ca, Patricia Odero, Ahmed Bashir, Serge Manituo Somda, Fatuma Adan, Martin Weiss, Wayne Naidoo, Frank Adjei Benin, Todd Malone, Christopher A. LeGrand, Andrew Yona Kitua

**Affiliations:** a BroadReach Health Development; b West African Health Organization (WAHO); c Duke University Global Health Innovations Center; d Intergovernmental Authority on Development (IGAD); e Jembi Health Systems

## Abstract

The emergence of COVID-19 highlights globalisation realties, where diseases may emerge from anywhere and rapidly spread globally. Lessons emphasise the necessity for strengthening regional and global collaboration and coordination to allow rapid risk identification, resource mobilisation and joint actions. We report the experience of the Regional Action through Data (RAD) partnership in fostering regional cooperation and collaboration to use data for battling infectious diseases and the effects of COVID-19. The Partnership comprised;BoadReach company, The West African Health Organization (WAHO) and the Intergovernmental Authority on Development (IGAD); Duke University Global Health Centre and the Jembi Health Systems, South Africa.

**Main objective:**

To address the problem of limited used of data to drive performance in healthcare service delivery in sub-Saharan Africa; by changing how and why data is collected, analysed, and then used to achieve results.

**Specific objectives:**

1. Regional level: To equip and empower IGAD and WAHO with evidence-based analytics to drive data use for evidence-based policy and program action in public health (regional level). 2. Patient-provider level: To deploy and implement a digital health solution for child-hood vaccination services focused on mobile cross-border populations along the Uganda-Kenya border.

Engagement approaches used included; meetings, workshops, technical working groups, establishing monitoring system and annual implementation revision. Targeted training and capacity building were conducted. All activities were built on existing systems and structures to strengthen ownership and sustainability.

**Regional level achievements:**

1. Regional health data sharing and protection policy, 2. Strengthened regional health information platform. Patient provider level: Deployment of a cloud based digital health solution to enhance childhood access to vaccination services for cross border populations of Kenya and Uganda, 3. Both regions developed resource mobilisation plans for sustainability.

RAD established the foundation for building trust and strengthening regional collaboration and coordination in health in Sub-Saharan Africa.

## BACKGROUND

The frequent emergence of global health threats including the current COVID-19 pandemic highlights globalisation realties, where diseases may emerge from anywhere and rapidly spread across countries, regions, and continents.^1–3^ Experience from past threats plus the current COVID-19 pandemic emphasise the role of strong regional and global collaboration and coordination of efforts in containing and or preventing these threats at source.^1–5^ At the start of the 21^st^ century, Severe Acute Respiratory Syndrome (SARS) was identified early as a global threat and successfully contained through collaboration and coordination across countries.^1^ In contrast, unpreparedness at national and regional level to address disease outbreaks with potential for spreading rapidly globally led to the disastrous social and economic effects of the 2014-2016 Ebola outbreak in West Africa.^6–9^

Preparedness is a continuous process of implementing actions before, during and after any public health event.^7–9^ Preparedness is establishing active surveillance systems able to detect earliest signals of disease outbreak and alerting countries and regions to respond early and adequately. Countries and regions are required to invest in preparedness to have the capacity to mobilise adequate resources to prevent or contain disease outbreaks at source. Necessary capacities include; well prepared and resourced infrastructure i.e. hospitals and facilities for quarantine, sufficient workforce ready to take actions, sufficient stocks of personal protection equipment, medicines and other logistics as may be required during emergency. It further requires a strong disease surveillance system able to provide reliable data to inform policy, implementation, and practices fitting the moment (before, during or after outbreak).^7–9^

Maintaining functional country and regional public health infrastructures smoothens the implementation of public health interventions like quarantine and isolation of patients without delays. Such infrastructure must be adequately resourced to allow good patient care and ensure safety of health workers.^8–10^ Communities must be sensitised enough to understand the functions of such facilities and their protective values against disease spread among the community. Continuous advocacy and education build trust and prevents resistance to quarantine, isolation and other public health measures like contact tracing and safe burial. It is during peacetime that health personnel and social scientists may engage in educating communities and individuals to understand disease risks. This is the best time to advocate for effective interventions and change of practices that may fuel disease spread. It is the best moment to engage leaders and communities to change traditional practices that may fuel disease outbreaks and discuss alternative safe practices acceptable to the communities like safe burials.^8–10^ Communities and individuals need to be sensitised to understand and acknowledge their responsibilities towards preventing and addressing disease risks and to trust health workers as their helping hands. Preparedness further means anticipating health threats and their worst situations. This requires regular workforce rehearsal in the form of simulation exercises to remain ready to respond at any time.^9^ Regional preparedness means being ready to continuously share data and be informed early of any impending health threats. It further means readiness to mobilise and/or share resources rapidly, devise and implement evidence informed strategies to contain any outbreak at source. Ability and readiness to help a member state contain an outbreak at source is critical to prevent regional health threat. Regional preparedness also calls for fostering the culture of using data for actions and practices which require continuous evidence informed dialogue on disease prevention and control.^7–9^ Regions should ensure availability of updated contingency plans and readiness to apply such plans at any moment when alerted. Countries are continuously reminded of their obligation to invest in building the core capacities outlined in the International Health Regulations (IHR) 2005.

Most of these preparedness aspects were missing during the 2014-2016 Ebola outbreak in West Africa.^8–10^ It took some time to recognise it as a regional and global threat following initial alerts. Countries acted in silos and in confusion and their efforts lacked multi-sectoral collaboration and coordination. Even the global community delayed in recognising and declaring global emergency thus delaying regional and collaborative actions. Lack of adequately prepared infrastructure and resources led to panic and putting of health workers at high risk. Lack of trust and community engagement led to community panic and spread of conspiracy theories including refusal to abide by public health measures like safe burials. Evidence based and effective interventions were resisted, and health workers were attacked.^3–5^

The West African leaders came to recognise this problem and made commitments to strengthen collaboration to strengthen national and regional preparedness.^11–12^

Following such recognitions, regional and global efforts were made to help countries prepare better for the next outbreak/pandemic. This included the launch of the Emerging Pandemic Threats Programs 1 (EPT-1) and 2 (EPT-2)by USAID.^13,14^ The programs aimed at strengthening developing countries' capacities to prevent, detect and control infectious diseases in animals and humans in recognition of the human-animal pathogens interphase. Projects under EPT-1 included; Predict, Identify, Prevent and Respond. Projects under EPT-2 were Predict-2, One Health Workforce, and Preparedness and Response (P&R). Among the achievements of the P&R project was the establishment of functional Multi-Sectoral Coordination (MCM) Mechanisms in Africa.^15,16^

As a result, when the next Ebola outbreak happened in 2018, improved regional and global collaboration, coordination, resource mobilisation and understanding of community perceptions and needs favoured its containment in the Democratic Republic of the Congo (DRC). It also fast tracked the development, testing and deployment of an effective vaccine.^16,17^ Currently, effective global collaboration and coordination produced a vaccine for COVID-19 in a shorter than expected period, thus emphasising the power of collaboration and joint coordinated actions.^2,3^

In this study, we report the experience and achievements of efforts to foster regional cooperation and collaboration for battling infectious diseases. We also discuss the effects of COVID-19 on the project. This study was conducted by a partnership called Regional Action through Data (RAD). The RAD partnership formation was facilitated by the United States Agency for International Development (USAID) by convening health technical experts from Africa and USAID to identify pressing African health needs and their solutions. Inadequate use of data to drive health policies and practices in Sub-Saharan Africa was identified as among the needs. The partnership involves the BroadReach company as the prime (providing oversight and technical support), the West African Health Organization (WAHO) of the Economic Community of West African States (ECOWAS) and the Intergovernmental Authority in Development (IGAD) of the Horn of Africa. It also involved 2 technical partners namely; the Duke University Global Health Innovation Centre (experts in data governance and protection) and the Jembi Health System a non-profit organisation working on digital health systems for Africa low-resource settings.

The main objective of RAD partnership was to address the problem of limited used of data to drive performance in healthcare service delivery in sub-Saharan Africa; by changing how and why data is collected, analysed, and then used to achieve results.

The specific objectives were: 1. Regional level: To equip and empower IGAD and WAHO with evidence-based analytics to drive data use for evidence-based policy and program action in public health.

2. Patient/provider level: To deploy and implement a digital health solution for child-hood vaccination services focused on mobile cross-border populations along the Uganda-Kenya border; for continuity of vaccination services regardless of their location

## METHODS

The RAD project's approach was to engage the regional Organisations (WAHO and IGAD) and their respective member states to participate actively throughout the project's life from identifying the idea/s, setting the objectives and through implementation. The project provided technical and financial support, while the activities were implemented by the regions and member states. The implementation was therefore institutionalised within the regions' health information systems, thus enhancing capacity building and ownership.

**FIGURE 1.**
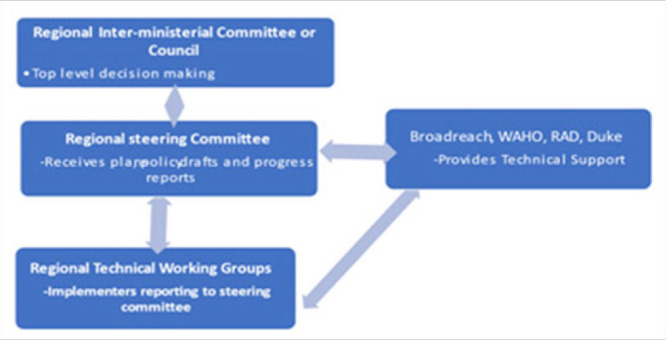
The RAD Partnership Organisation Structure

The RAD partnership structure was therefore constructed to allow two-way flow of information and dialogue between the project's implementing technical, regional and national program managers and the top-level regional decision makers, the ministries of health. See figure 1

The initial steps included holding a project initiation meeting between technical experts and regional program managers of health to have the approval to conduct the project in alignment with regional needs. The meeting also served to define the projects' governance structures. A similar governance structure was adopted in both regions as shown in figure 1. At the apex, the projects were linked to the regional inter-ministerial committee for health which is the regional decision maker on health. This link was provided through the projects steering committee formed by health managers who report and receive directives from the inter-ministerial committee/council (Ministers of health of member states). The steering committee oversees the overall management of the project, receives performance and progress reports and reviews proposed plans and policies before submitting them to the inter-ministerial committee for approval and ratification as regional plans and policies. The steering committee has technical working groups for the different lines of actions under the project objectives. These are comprised of selected technical experts from WAHO and member states. They are the implementers of the activities. They report and receive directives from the steering committee. Financial and technical support is provided by Broadreach. Technical support to for the establishment and implementation of regional health sharing and protection policies is provided by the Duke University, while technical support to strengthen cross border populations' access to health services focused on enhancing childhood vaccination is provided by Jembi Health Systems, a Non-Profit Company based in South Africa.

To facilitate member states' buy in and enhance projects' advocacy, the technical working groups present their work at regionally organised Annual Health Information meetings with regional health program managers. From these meetings, comments/inputs are gathered which are used to come up with draft reports, plans and proposals for policy or practices before they are submitted to the steering committee. These meetings also receive reports of project progress and achievements.

Addressing the regional level targeted specific objective involved the following steps. In the WAHO region, initial discussions for building consensus and getting approval to conduct the project were conducted at the regional joint Health Management Information System (HMIS), Integrated Disease Surveillance and Response (IDSR), and One Health data managers' meeting held on July 2019, in Abuja, Nigeria. In August 2019, preparations for development of the WAHO institutional data governance and protection policy started. A consultant was engaged to conduct a survey to determine the level of data use in WAHO, data lifecycle from collection through storage, use and sharing. This involved field visits to WAHO offices involved in health data governance (Information Technology (IT), administration, finance, projects' management, and health programs implementation).

The draft policy document was subjected to several rounds for review through member states consultations before it was validated at the 10^th^ ECOWAS HMIS meeting in March 2020, Banjul, Gambia. The WAHO institution uses this meeting for both technical groups presentations and to submit documents to the Steering committee formed by HMIS national managers. The document was then handed to the WAHO institution legal board for finalisation with legal language formatting. Subsequently it was ratified by the Assembly of Health Ministers of ECOWAS and by the Council of Heads of States as a regional policy.

The Duke University Global Health Centre conducted a survey to identify gaps in data governance at national level that needed alignment with the regional health data governance and level of alignment to the regional data governance policy. The survey results and suggestions for aligning national data governance policies to the WAHO policy were shared with member states. A webinar to allow open discussions of the results among member states and build consensus on alignment approaches was planned for October 2021.

Through discussions and negotiations, WAHO demanded the strengthening of regional health information products to enable the region to track and address emerging infectious diseases outbreaks. This was driven by the COVID-19 pandemic as the region felt the urgency for quality data to inform regional policies, decisions, and guide practices.

RAD employed 3 technical staff to support WAHO to review the quality and regular production of existing health products. Revealed gaps in data quality, collation from different sources and irregular production pushed the development of a regional COVID 19 dashboard. A test dashboard was immediately developed and presented to the Health Management Information System (HMIS), Integrated Diseases Surveillance and Response (IDSR) and One health data managers during a virtual meeting. The meeting approved the dashboard developedand recommended the production of a weekly disease epidemiology bulletin, a quarterly epidemiological bulletin, and an annual regional disease profile. In addition, the regional Director demanded the production of an annual regional disease profile and a 5years regional disease profile. The RAD project supported the regional team through the newly employed technical staff to train countries to collate data from different sources and provide robust regional data. A regional health data platform was also refurbished with new equipment and technology updates to receive and store regional health data. A similar process, with exception of employing technical staff, was followed in addressing the same objective in the IGAD region.

The second specific objective was only implemented in the IGAD region. A baseline analysis was conducted to identify sites for testing and deploying the digital health solution for child-hood vaccination services focused on mobile cross-border populations along the Uganda-Kenya border, for continuity of vaccination services regardless of their location. 4 sites were identified based on the selection criteria which included; 1. Being served by a large mobile population, 2. Willingness to enter into an agreement to share immunisation data across borders 3. Trainable staff willing to collect data using special mobile phones and upload the data into data servers. Data servers were placed in safe positions within the facilities and a selected staff engaged in childhood immunisation data collection were equipped with mobile phones. Jembi health systems technical experts accompanied by Broadreach's technical IT expert visited the sites and deployed the server equipment at the facilities. They trained the facility nursing staff on the use of the mobile phones for data collection and appointed an administrator per facility to upload the data into the servers on a regular basis. Training was repeated annually to keep the staff updated. Ad hoc training was conducted whenever there were staff transfers to bring on board new staff. Regular quarterly site supervision visits were conducted to ensure staff morale and motivation while checking the conduct of project activities. Trouble shooting was conducted through the site supervisory visits and virtually in collaboration withJembi health systems technicians and WAHO local IT personnel.

**FIGURE 2.**
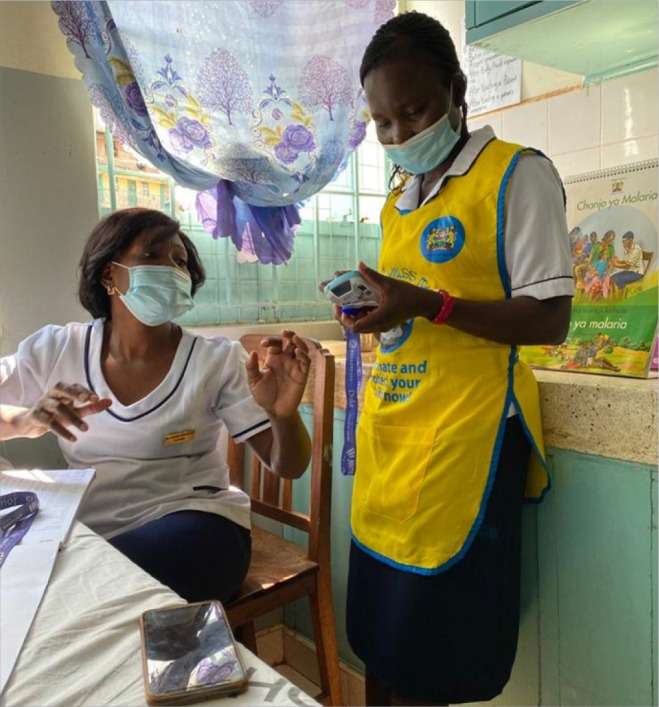
Nurses Practicing how to Access Journey Solution Stored Health Information

**FIGURE 3.**
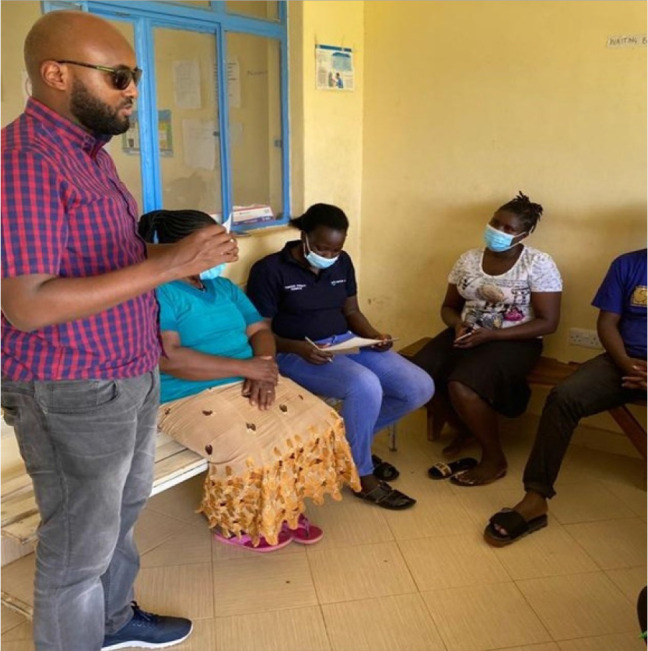
Training Session on Journey Solution Data Entry and Upload

Mothers participating in the childhood immunisation programs were provided with electronic cards that store the vaccination information of their children. These cards can be read in any cross-border facility where the Journey solution was available. Figure 2 & Figure 3

IAD Cross-Border Immunisation. By Ahmed Bashir-2020

IAD Cross-Border Immunisation. By Ahmed Bashir-2020

In general, before regional health data governance and protection policies were enacted, RAD engaged with member states and implementers through the signing of special agreements (Memorandum of Understanding (MoUs) to allow implementation and official recognition of project's activities.

The consortium created and applied a strong monitoring and evaluation system which included weekly activity - implementation reviews by the Broadreach team; Biweekly individual review with specific implementers like those involved in the production of weekly epidemiological bulleting or deployment of the Journey solution; Monthly project implementation updates with individual partners (WAHO, IGAD, DUKE and Jembi); Monthly progress updates to USAID; Quarterly consortium meeting to review progress and sharing experiences (joint BroadReach, WAHO, IGAD, DUKE and Jembi); Quarterly reports and annual reports. Annual project reviews were conducted to guide new year's activities planning, setting timelines, and budgets. Each partner reviewed their plans and submitted a fresh annual implementation plan for the new year.

## RESULTS

In the fifth and final year of implementation, RAD achieved its key objectives and related deliverables. These include: 1. Regional data governance policies for WAHO and IGAD regions – Allowing regional health data sharing, 2. Quality enhanced health data sharing tools (regional health information products). 3. Cloud based Personal Electronic Health Record System (Mobile electronic health records) -to ensure quality and continuity of child health care (vaccination) regardless of their location. A summary of the achievements is provided in Tables 1 and 2.

**TABLE 1:**
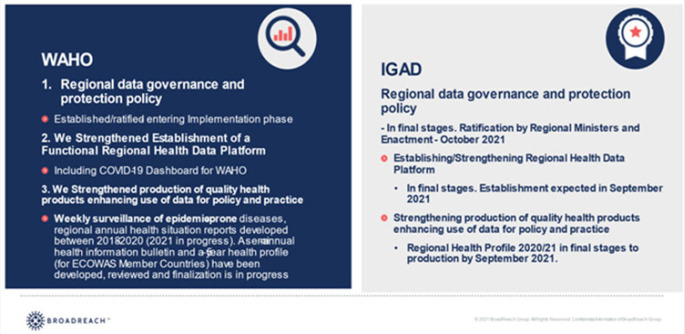
Regional Levels Achievements: Building a Culture of Data Use for Policy and Decision

**TABLE 2:**
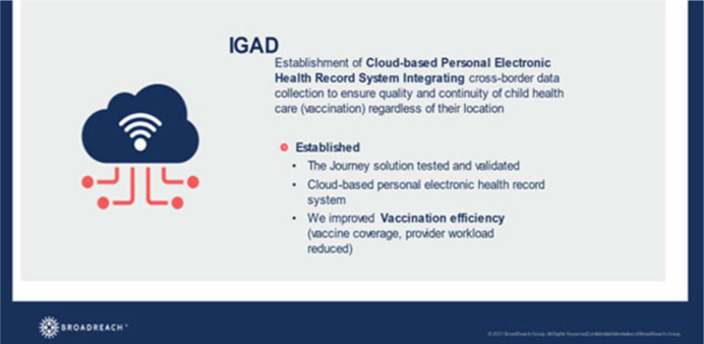
Community Patient Provider Level Achievements: Enhanced Access to Health Services for Cross Border Populations

### Additional Achievements:

**FIGURE 4.**
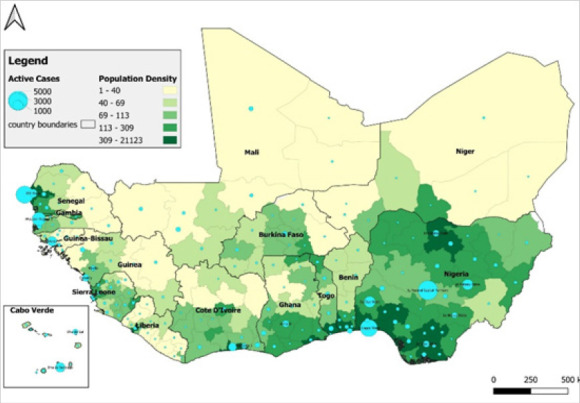
Geographical Distribution of COVID-19 in the ECOWAS.

**FIGURE 5.**
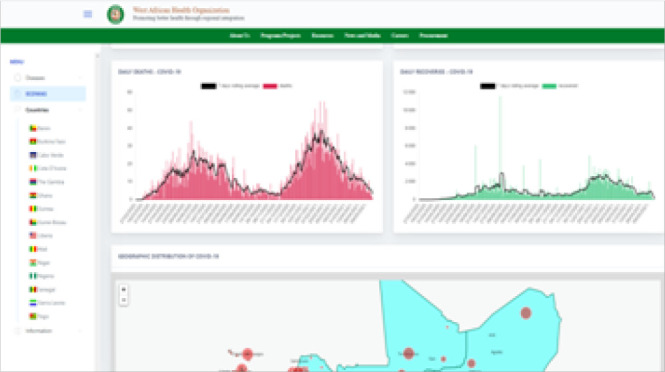
Screenshot of the WAHO COVID 19 Platform

Both regions are sharing disease surveillance data and driven by the urgency for addressing the COVID-19 pandemic, they have established functional COVID-19 dashboards.^18,19^ The WAHO dashboard was developed with RAD support while the IGAD dashboard was developed by IGAD member states with support from the World Health Organization. Extracts from WAHO's COVID 19 Dashboard is presented in figure 4 and 5.

**FIGURE 6.**
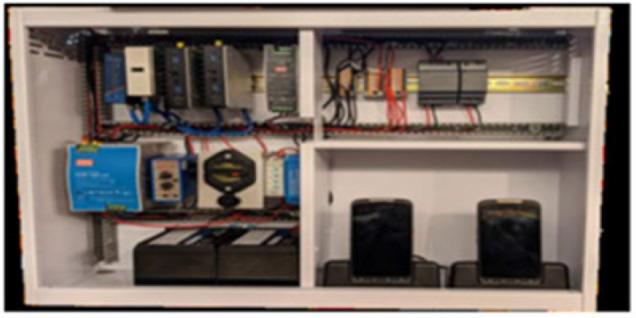
The Cloud Based Personal Electronic Health Record System Server Box

**FIGURE 7.**
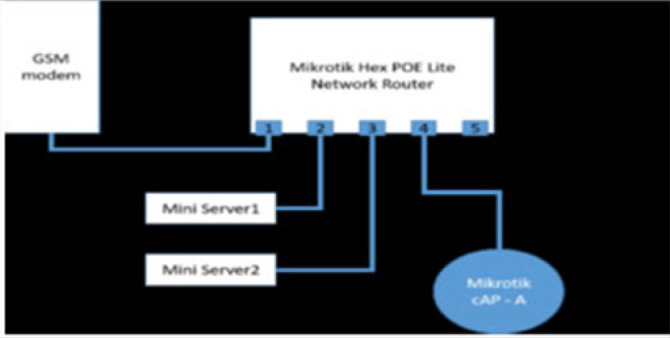
The Cloud Based Personal Electronic Health Record System Components' Links

The Cloud based Personal Electronic Health Record System (Mobile electronic health records) server system using the Open Horizons technology is illustrated in Figure 6 and 7.

The Journet Solution Sever Box. The unit consists of a metal chassis with several electronic components mounted within the chassis and secured via NS35 DIN rails and cable management conduits

The Server Components “Open Horizons” Technology

Currently, 4 facilities along the border between Kenya and Uganda are fully operating the cloud based Personal Electronic Health Record System in 4 health facilities.

The system will be established in 2 additional facilities along the same border by end of October 2021. Around 20,000 personal cards have been issued since 2018, covering 140,000 immunisation events. Data has indicated patient retention rate of 97%. Only 100 cards were replaced since the system began operating in 2018. 23 health workers have been trained to manage the tools and data upload and additional staff training is planned.

### COVID-19 Effects

The COVID-19 pandemic took everyone by surprise. Neither the RAD partnership nor the regional organisations were ready for it. The pandemic posed the following challenges: 1. Delays in implementation of planned activities which required person to person meetings. 2. Additional pressure on the health systems on which RAD activities were being implemented. 3. Deviated attention by regional leaders towards the pandemic and less to projects like RAD. 4. Introducing a new regional priority to the fight against diseases. The situation was worsened by the already year 4 (2019-2020) project's budget cuts whereby the partnership was already operating under financial constraints. Undesirably, due to inadequate preparedness of the RAD partnership and member states, the implementation of RAD projects activities slowed down for about 4 months (January-April 2020). The required continuous engagement with the partners became cumbersome and slow. Actions which required decisions by regional leadership towards the projects' activities implementation took longer than usual. Fortunately, after 4 months of uncertainty and activities slow down, the RAD partnership recovered. It slowly began implementing innovative strategies to allow continuity of activity implementation. The new strategies included conducting activities virtually. All meetings were conducted virtually. Engagements with partners' representatives from member states became virtual and it became necessary to organise several smaller groups than large single group discussions with the virtual approach for better results. Field supervisory visits were conducted virtually and so were trouble shooting activities related to the operations of the cloud based personal health records system for childhood immunisation. However, the additional pressure on health services and regional leadership became an opportunity to support the health system to address the pandemic. The COVID-19 situation posed need for strengthening regional collaboration and coordination. The WAHO regional leadership demanded the production of quality health products and facilitated engagements and collaboration among different health programs working towards the same goals. The RAD partnership was flexible enough to allow adjustments of its activities to support regions to respond to the COVID-19 emergency needs. Improved collaboration between the Integrated Disease Surveillance and Response, One Health Multi-sectoral Coordination Mechanisms and Health Management Information Systems facilitated the production and use of health information products. The production and use of the weekly disease epidemiology bulleting improved to the extent that it was transferred to be under the ECOWAS management to enhance its use at regional level. The COVID-19 dashboard came about as a special demand from the WAHO leadership and as soon as it went live, countries began demanding assistance to establish their national dashboards. The same urgency pushed IGAD to develop its own COVID-19 dashboard in partnership with the World Health Organization

The application of the COVID-19 Best practices had also 2 sides. On one hand, workers were not used to wearing masks, hand washing and frequent use of sanitisers when attending their office duties. Therefore, it took a while to achieve full compliance. Broadreach applied social distancing at work and by restricting access to office to only needed workers. The staff were instructed to work from home. This deprived staff the usual social company they were used to. It was therefore stressful to miss the face-to-face contacts and be glued to one's computer at home for the working hours because this became the working tool. Broadreach therefore introduced regular “Be well” sessions to help people learn how to cope with stress. Feedback from staff during these sessions is that they werevery helpful. Work performance does not appear to have been affected negatively by COVID-19 Best practices, but to maintain good work performance meant conducting of frequent tele-conferences and meetings, which were not compatible with working from home. Working from home requires time to spent with family members, Children demand extra attention which conflicts with work demands. On the other hand, working from home offered the opportunity to spend more time with beloved ones and also opportunity to help with family chores. The conflict between work and home demands may have added stress to those who could not cope well and again the “Be well “sessions including sessions on mental health became very useful. In general, when talking to staff members, it would appear that the worst has passed, but staff continue to miss each other's presence and the social interactions working together in an office gives. The limitations of this section on best practices islimited research.

## DISCUSSION

Both regions have regional health data governance and protection policies (IGAD's policy is for ratification by the regional inter-ministerial committee in November 2021). To facilitate implementation of the policies, user-friendly shorter versions of the policy documents were developed. In addition, each region has been availed with a guide to use the policy document. The establishment of regional health data governance and protection systems in 2 economic regions of Sub-Saharan Africa was a breakthrough in enhancing regional collaboration and coordination of efforts to fight infectious diseases and other health threats. It is a significant achievement in that the 2 regions (WAHO and IGAD) have legal instruments allowing for formal discussions and formulation of regional health policies and practices which are informed by quality regional data. The need for stronger regional collaboration and coordination of efforts has been recognised by the regional leaders, but it required legal instruments to support actions.^7^ The 2 regions were encouraged to start sharing data even before the data governance policies were ratified. This helped to maintain continuous dialogue that allowed clarifications and trustbuilding. The implementation of the ratified policies is therefore a continuity of regional leadership initiated and led practice. For example, while all WAHO member states are currently sharing data with the regional - health information platform, the transfer of data has been conducted manually to upload data from server to server. Now having established the data governance and protection policy, WAHO has initiated and is progressing well with arrangements to establish automatic data transfer (sharing) from country data servers to the regional health data server. 2 countries; Sierra Leone and Guinea have already received technical support, completed negotiations and are transferring automatically national health data to the regional server. 3 more countries including Ghana, Liberia, and The Gambia, are in the process of doing so while negotiating and building trust with other member states continues.

The IGAD region was slower in implementing RAD activities but is following closely on a similar path of collaboration and data sharing. The regional data server has already been secured and it is in the process of being deployed to serve the IGAD regional health information platform. Its regional data sharing policy is ready for submission to the Inter-ministerial committee for ratification and endorsement. The IGAD's slow pace was caused by the fact that the 2 regions entered the RAD partnership at different stages of development towards regional data sharing. The WAHO region entered the partnership having initiated its own regional health information platform and member states were better informed and prepared to move towards regional data sharing. Importantly the IGAD region manage to initiate bilateral agreements (Memorandum of understanding) to allow data sharing while developing the regional health data governance and protection policy. For example, through such arrangements it was possible to deploy the cloud based Personal Electronic Health Record System and strengthening cross border children immunisation programs of Kenya and Uganda.

## CONCLUSIONS AND RECOMMENDATIONS

As pronounced by African wisdom “If you want to go fast, go alone. If you want to go far, go together”, the RAD journey required patience and strong collaboration to maintain focus, momentum, and build trust among partners. Through RAD, we learn that nurturing regional collaboration and coordination for effective health solutions takes long and requires patience. Projects or programs of this nature should be long term and should also consider a phased handover period to allow maturity and sustainability of initiatives. We also learn that partnerships and regional institutions need to always be prepared to adjust quickly and respond to shocks. In addition, projects need to have flexibility to be able to adjust and respond to regional and country needs especially at times of emergencies. It is important for partnerships to support regions and countries to respond to emerging needs at times of emergency. That flexibility enhances trust building and adds value to the presence of the project. Furthermore, we learn that health emergencies can be opportunities for strengthening the weak health systems since they create demand to the health authorities. Projects and partnerships must have an eye for and be better prepared to seize such opportunities.

RAD established the foundation for building trust and strengthening regional collaboration and coordination in health in Sub-Saharan Africa. This foundation has demonstrated great potential for fighting infectious diseases outbreaks including the COVID-19 pandemic. RAD also planted the seeds for a culture of data use to inform policy and decisions in Sub-Saharan Africa. The WAHO and IGAD regions need to implement their resource mobilisation plans to secure internal and external resources to maintain and build on RAD's gains. Regions and countries shouldinvest adequately in strengthening and building on this foundation to strengthen their response to COVID-19. Such investment will have spill over benefits if used as an opportunity to strengthen regional and national preparedness to address the next pandemic.
